# Isolation and Quantification of miRNA from the Biomolecular Corona on Mesoporous Silica Nanoparticles

**DOI:** 10.3390/nano11051196

**Published:** 2021-05-01

**Authors:** Carla Vidaurre-Agut, Eva María Rivero-Buceta, Christopher C. Landry, Pablo Botella

**Affiliations:** 1 Instituto de Tecnología Química, Universitat Politècnica de València-Consejo Superior de Investigaciones Científicas, Avenida de los Naranjos s/n, 46022 Valencia, Spain; carviag@itq.upv.es (C.V.-A.); evribu@upvnet.upv.es (E.M.R.-B.); 2 Instituto de Instrumentación para Imagen Molecular (I3M), Centro Mixto CSIC-Universitat Politècnica de València, Camino de Vera s/n, 46022 Valencia, Spain; 3 Department of Chemistry, University of Vermont, 82 University Place, Burlington, VT 05405, USA

**Keywords:** mesoporous silica nanoparticles, biomolecular corona, miRNA, miR-200c, miR-221, miR-375, prostate cancer, cancer diagnostic

## Abstract

To understand the factors that control the formation of the biomolecular corona, a systematic study of the adsorption of several miRNAs shown to be important in prostate cancer on amine-functionalized mesoporous silica nanoparticles (MSN-NH_2_) has been performed. Process parameters including miRNA type, nanoparticle concentration, incubation temperature and incubation time were investigated, as well as the potential competition for adsorption between different miRNA molecules. The influence of proteins and particle PEGylation on miRNA adsorption were also explored. We found that low particle concentrations and physiological temperature both led to increased miRNA adsorption. Adsorption of miRNA was also higher when proteins were present in the same solution; reducing or preventing protein adsorption by PEGylating the MSNs hindered adsorption. Finally, the amount of miRNA adsorbed from human serum by MSN-NH_2_ was compared to a commercial miRNA purification kit (TaqMan^®^, Life Technologies, Carlsbad, CA, USA). MSN-NH_2_ adsorbed six times as much miRNA as the commercial kit, demonstrating higher sensitivity to subtle up- and downregulation of circulating miRNA in the blood of patients.

## 1. Introduction

The interaction of molecules from biological samples (e.g., from blood) with nanoparticles promotes a series of processes leading to the formation of the so-called “biomolecular corona” [1,2,3,4,5,6,7], a multilayered mass of biomolecules including proteins, RNA, lipids, carbohydrates that adsorbs from solution onto the surface of the nanoparticles. A few years ago, the concept of using the “personalized biomolecular corona” (PBC) as a pattern linked to physiological conditions was introduced as a potential tool for the early diagnosis of cancer and other diseases [8,9]. Since then, various authors have applied this concept to detect specific pathological stages of disease [10,11,12,13]. In this context, efforts have been made to isolate and identify specific proteins or clusters of proteins that could be used as biomarkers [14]. 

MicroRNAs (miRNAs) are small regulatory RNAs present in blood in stable forms (e.g., exosomal miRNAs and protein complexes) and have been identified as noninvasive biomarkers of molecular changes associated with cancer [15,16,17], showing promise as signatures for cancer detection and prognosis. Nevertheless, the analysis of extracellular miRNAs from biological fluids is severely affected by sensitivity issues. Isolation and accurate quantification of miRNAs from among the broad range of serum molecules is challenging due to the small size of miRNAs and high levels of contaminating moieties. These limit the efficacy of standard RNA extraction protocols and commercially available kits and their application for diagnostic purposes [18]. Tailored platforms such as the Scano-miR bioassay [19,20] have shown good performance in the detection of circulating exosomal miRNA in serum samples of cancer patients with specific disease prognosis (e.g., low risk or high risk), but there is plenty of room to develop broader spectrum clinical technologies. 

Prostate cancer (PCa) is the most commonly diagnosed malignancy in men in the United States and in elderly men in Europe [21,22]. Early detection of clinically localized PCa is the most efficient approach to reduce unnecessary treatments and decrease deaths, and serum prostate specific antigen (PSA)-based testing has been widely used in the last decades for PCa detection. Unfortunately, serum PSA can be elevated due a number of reasons that do not involve cancer such as benign prostate hyperplasia (BPH), and overdiagnosis and overtreatment occur frequently, involving serious side effects and unnecessary clinical expenses. Conversely, miRNAs have been selected as important regulators of tumor progression in PCa and they can be used to accurately distinguish between carcinoma and BPH, and can also be used to identify molecular signatures associated with patients with aggressive PCa [20,23,24]. In particular, circulating miR-200c, miR-221 and miR-375 are up-regulated in PCa, and have been proposed as potential biomarkers [20,23,24]. 

To our knowledge, no previous investigation on the analysis of miRNA from blood biomolecular corona has been carried out. Indeed, the only paper exploring its diagnostic possibilities refers to miRNA determination by using magnetic nanoparticles (MNPs) functionalized with carboxylic acids to collect and enrich proteins in urine and cell culture media [25]. That work points out that miRNAs are frequently associated with specific serum protein complexes for protection from digestion by RNases. Considering this fact in the context of our previous work on the protein coronas of PCa patients prompted us to investigate the possibility of isolating specific miRNA moieties from serum samples. In this study, we have isolated the miRNAs miR-200c, miR-221 and miR-375 molecules from the biomolecular corona developed over amino-modified mesoporous silica nanoparticles (MSN-NH_2_), monitoring adsorption process variables, such as concentration, time, temperature, surface functionalization (e.g., PEGylation) and the presence of proteins, and quantifying the miRNa profile. We also have compared the efficacy of this novel miRNA isolation and quantification protocol with a commercial miRNA purification kit (TaqMan^®^) over standard human serum samples spiked with miR-200c, miR-221 and miR-375 sequences. The ultimate goal is to develop an early diagnostic test based on miRNA expression patterns, and in this context, we show that the use of nanoparticles as nano-concentrators able to isolate miRNAs as components of the biomolecular corona shows significant potential.

## 2. Materials and Methods

### 2.1. Reagents and Standards

Duplex sequences of miR-200c, miR-221 and miR-375 were provided by Integrated DNA Technologies (IDT), whereas proteins complement C3 and apolipoprotein AII were supplied by Athens Research and Technologies (ART). Other reagents and solvents were purchased from Sigma-Aldrich (Munich, Germany) unless otherwise noted, and used as received. Synthetic protocols corresponding to preparation of mesoporous silica nanoparticles (MSNs), as well as surface modification with organic ligands are fully detailed in the Supplementary Information. Techniques used for physico-chemical characterization of MSNs are also described in the Supplementary Information.

### 2.2. Isolation and Quantification of miRNAs from Biomolecular Corona

#### 2.2.1. General Protocol for Isolation of miRNA

**Figure 1:**
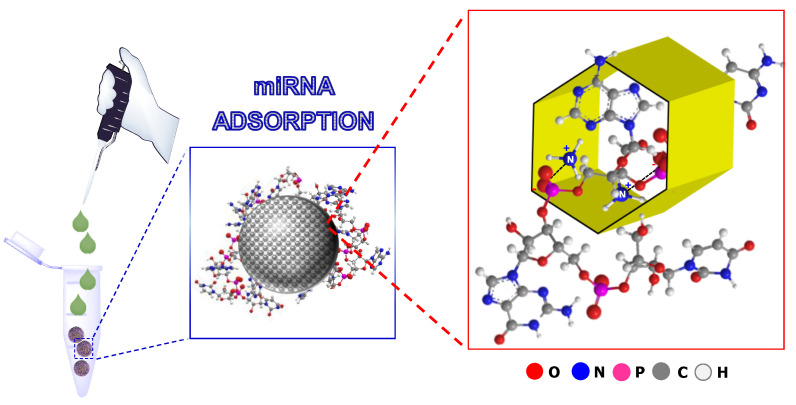
Artistic scheme of the miRNA adsorption process on MSN-NH_2_.

The amount of 0.5 mg MSN-NH_2_ was put in a 1.5 mL Eppendorf tube and 500 µL nuclease free water and 5 µL miRNA (~0.6 mg/mL solution) was added. The sample was vortexed and typically incubated for 1 h at 37 °C and 1500 rpm in a Thermomixer^®^ unit (Eppendorf, Hamburg, Germany). Afterwards, the nanoparticles were centrifuged (5 min, 14,800 rpm) and separated the supernatant. Subsequently, nanoparticles with miRNA corona were treated in a 1.5 mL Eppendorf tube with 400 µL nuclease free water and 33 mg (0.40 mmol) sodium acetate. The sample was vortexed and incubated for 30 min at 50 °C and 1500 rpm in a Thermomixer^®^, and further centrifuged (5 min, 14,800 rpm). Then, the supernatant was separated and 1 mL of cold ethanol was added, followed by incubation 30 min in ice. This miRNA suspension was centrifuged (15 min, 14,800 rpm, 1 °C) and the pellet was isolated, freeze-dried (−55 °C, 16 h) and later reconstituted with 50 µL nuclease free water, and the adsorbed miRNA was determined at 260 nm using a NanoDrop^TM^ ND1000 spectrophotometer (Thermo Fisher Scientific, Wilmington, DE, USA). All experiments were done in triplicate. An artistic representation of this process is presented in Figure 1.

#### 2.2.2. Influence of Process Parameters in Formation of the miRNA Corona

The influence of different process parameters on the miRNA corona formation was determined by the adsorption of a single miRNA molecule on MSN-NH_2_. The following variables were investigated: miRNA type (miR-200c, miR-221 or miR-375), nanoparticle concentration (1, 2 or 4 mg/mL; miR-221), incubation time (15, 60 or 120 min; miR-375), and incubation temperature (25, 37 or 50 °C; miR-375). The used protocol was the same as above unless specific process conditions. All experiments were performed in triplicate.

#### 2.2.3. Adsorption of Multiple miRNAs

The potential interactions between different miRNA molecules and their effects on the formation of miRNA corona was studied by adsorption of two or three miRNA types on MSN-NH_2_.

For the study of two miRNA adsorption, 0.5 mg MSN-NH_2_ was put in a 1.5 mL Eppendorf tube and 500 µL nuclease free water, 5 µL miRNA_a_ (~0.6 mg/mL solution) and 5 µL miRNA_b_ (~0.6 mg/mL solution) was added. The sample was vortexed and incubated for 1 h at 37 °C and 1500 rpm in a Thermomixer^®^ unit. Afterwards, the nanoparticles were centrifuged (5 min, 14,800 rpm). Subsequently, nanoparticles with miRNA corona were treated in a 1.5 mL Eppendorf tube with 400 µL nuclease free water and 33 mg (0.40 mmol) sodium acetate. The sample was vortexed and incubated for 30 min at 50 °C and 1500 rpm in a Thermomixer^®^, and further centrifuged (5 min, 14,800 rpm). Then, the supernatant was separated and 1 mL of cold ethanol was added, followed by incubation 30 min in ice. This miRNA suspension was centrifuged (15 min, 14,800 rpm, 1 °C) and the pellet was isolated, freeze-dried (−55 °C, 16 h) and later reconstituted with 50 µL nuclease free water, and the adsorbed miRNA was quantified at 260 nm using a NanoDrop^TM^ ND1000 spectrophotometer. 

To determine the miRNA distributions in the mixtures, the freeze-dried miRNA suspensions were reconstituted in 100 µL nuclease-free water rotating at 4 °C for 1 h. Concentrations at this stage were determined on a NanoDrop^TM^ 2000 spectrophotometer (Thermo Fisher Scientific, Wilmington, DE, USA). Either 150 ng (Group I—same RNA mass) or 2.5 µL (Group II—same RNA volume) RNA was used as input to the miScript RT cDNA synthesis kit (Qiagen) following the manufacturer’s recommended protocol. The miSCript SYBR Green PCR kit (Qiagen) was used to interrogate individual miRNA on a ViiA 7 Real-Time PCR System (Applied Biosystems, Waltham, MA, USA) following the manufacturer’s protocol using 0.1 µL cDNA per 10 µL qPCR reaction in a 384-well plate, with triplicate wells for each cDNA/standard. The included Universal Reverse Primer was used with miRNA-specific forward primers:

miR-200c-3p: 5′–TAATACTGCCGGGTAATGATGGA–3′

miR-221-3p: 5′–AGCTACATTGTCTGCTGGGTTTC–3′

miR-375-3p: 5′–TTTGTTCGTTCGGCTCGCGTGA–3′

A 5-point serial dilution (1:10–1:10^5^) was used to generate a standard curve from a mixture of 2 µL cDNA synthesized (both Groups I and II) from MSN samples incubated with all three miRNAs (six total cDNA samples). Wells with poor amplification and/or melt curves were excluded. Ct values, relative quantity, and standard deviation were calculated in the QuantStudio^TM^ software (Thermo Fisher Scientific, Wilmington, DE, USA) and plotted in Prism 8 (GraphPad, San Diego, CA, USA).

Three different two-component miRNA mixtures were analyzed in this manner: miR-200c + miR-221; miR-200c + miR-375 and miR-221 + miR-375. An additional analysis was performed using a three-component miRNA mixture (miR-200c + miR-221 + miR-375). In the three-component adsorption experiment, 0.5 mg MSN-NH_2_ was put in a 1.5 mL Eppendorf tube and 5 µL of every miRNA (0.6 mg/mL solution) and 500 µL nuclease free water was added, and the procedure from this point was identical to that for the two-component mixtures described above. These experiments were done in duplicate.

#### 2.2.4. Influence of Protein Corona on miRNA Adsorption

The effect of proteins over the adsorption of miR-200c on MSN-NH_2_ surface was studied with two types of molecules, a small protein (apolipoprotein AII, Mw = 17 kDa), and a large protein (complement C3c, Mw = 138 kDa). For this purpose, several experiments were carried out. 

The amount of 0.5 mg MSN-NH_2_ was put in a 1.5 mL Eppendorf tube and 500 µL nuclease free water, 5 µL miR-200c (~0.6 mg/mL solution) and 40 µL protein AII or C3c (1 mg/mL solution) was added. Then, the sample was treated according to the standard protocol (Section 2.2.1), and the adsorbed miRNA was determined at 260 nm using a Nanodrop^TM^ ND1000 spectrophotometer.

Moreover, an experiment was performed by introducing both proteins at the same concentration: 0.5 mg MSN-NH_2_ was put in a 1.5 mL Eppendorf tube and 500 µL nuclease free water, 5 µL miR-200c (0.6 mg/mL solution), 40 µL protein AII (1 mg/mL solution) and 40 µL protein C3c (1 mg/mL solution) was added, and the analysis involved the steps described above. In addition, the influence of surface PEGylation over miRNA adsorption in the presence of both proteins was also validated by using PEGylated nanoparticles (MSN-PEG) with 2,5,8,11-tetraoxatetradecan-14-oic acid succinimidyl ester (Iris Biotech GMBH, Marktredwitz, Germany), and following the same protocol. All experiments were done in triplicate.

#### 2.2.5. Isolation of miRNA from Human Serum

In order to determine the efficacy of our protocol to isolate selectively miRNA molecules from real serum samples, we incubated our MSN-NH_2_ with human serum, and with human serum spiked with the three miRNA PCa biomarkers in concentration similar to that expected in real samples from PCa patients [26]. 

The amount of 0.5 mg MSN-NH_2_ was put in a 1.5 mL Eppendorf tube and 250 µL nuclease free water, 250 µL commercial human serum (male AB, Sigma-Aldrich, Munich, Germany), and 5 µL of every miRNA (~0.6 mg/mL solution) was added. The sample was vortexed and incubated for 1 h at 37 °C and 1500 rpm in a Thermomixer^®^ unit. Afterwards, the nanoparticles were centrifuged (5 min, 14,800 rpm). Subsequently, nanoparticles with miRNA corona were treated in a 1.5 mL Eppendorf tube with 400 µL nuclease free water and 33 mg (0.40 mmol) sodium acetate. The sample was vortexed and incubated for 30 min at 50 °C and 1500 rpm in a Thermomixer^®^, and further centrifuged (5 min, 14,800 rpm). Then, the supernatant was separated and 1 mL of cold ethanol was added, followed by incubation 30 min in ice. This miRNA suspension was centrifuged (15 min, 14,800 rpm, 1 °C) and the pellet was isolated, freeze-dried (−55 °C, 16 h) and later reconstituted with 50 µL nuclease free water, and the adsorbed miRNA was determined at 260 nm using a Nanodrop^TM^ ND1000 spectrophotometer. 

In addition, we compared the efficacy of our miRNA isolation system with a commercial TaqMan^®^ miRNA ABC purification kit. For this purpose, we followed the specification of the supplier (Life Technologies, Carlsbad, CA, USA). Firstly, 250 µL commercial human serum (male AB, Sigma-Aldrich, Munich, Germany), 250 µL nuclease free water and 5 µL of every miRNA (~0.6 mg/mL solution) were put in a 1.5 mL Eppendorf tube and vortexed for 30 s. A total of 50 µL of this soup was added to another 1.5 mL Eppendorf tube and 100 µL ABC buffer was introduced. Then, the mixture was vortexed again for 30 s. The process continued according to the manufacturer’s protocol (see Supplementary Information). The final freeze-dried was reconstituted with 10 µL nuclease free water, and the adsorbed miRNA was determined at 260 nm using a Nanodrop^TM^ ND1000 spectrophotometer. All experiments were done in triplicate.

#### 2.2.6. Statistical Analysis 

Data statistical analysis was performed using arithmetic means and error bars of statistical error means (SEM). The statistical analysis was carried out by Student’s t-test with a paired, two-tailed distribution, using the Prism 6 software (GraphPad, San Diego, CA, USA). Statistical significance was considered when *P* < 0.05 (*) or 0.01 (**).

## 3. Results and Discussion 

We have previously shown the influence of nanoparticle size in biomolecular corona formation, with small nanoparticles promoting molecule adsorption due to their favorable surface to volume ratio [6]. Therefore, we synthesized homogeneous, mesoporous silica nanoparticles (MSN-OH) with a particle diameter of about 45.0 ± 25.8 nm and a reasonable size range, as confirmed by dynamic light scattering (DLS, Table 1, Figure S1 in the Supplementary Information). Regular, monodispersed MSN-OH with a hexagonally ordered pore structure similar to that of MCM-41 (Table 1, Figure S2) were prepared by polymerizing silicate monomers in the presence of surfactant micelles [27]. BET-BJH measurements confirmed the type IV adsorption isotherm, with d_pore_ = 3.9 nm (Figure S3). However, the subsequent surface functionalization with amino groups (MSN-NH_2_) altered the crystallinity of the MSNs, and led to a reduction of the surface porosity and pore diameter due to partial pore blocking by the organic moieties. These impacts were larger for the PEGylated sample (MSN-PEG), as the bulky substituents may collapse pore entrances and channel interiors within the mesoporous structure.

TEM images showed that there was no significant particle size change when introducing amino or PEG groups (Figure S1 insets). In addition, the hydrodynamic diameter was not significantly compromised by amino group incorporation in MSN-OH, but the PEGylated nanoparticles were approximately 10% larger (Table 1). This increase is too large to be associated with the PEG molecule itself, and thus we attribute this change to the higher particle polydispersity associated with the PEGylation process. This was confirmed by the increase of the polydispersity index (PdI > 0.3), which was related to the formation of dimers and oligomers that enlarged the average particle diameter.

In addition, a noticeable reduction in the ζ-potential was observed in the functionalized materials (Table 1). The incorporation of amino groups with pKa > 9 (ionized approximately 95–99% as R-NH_3_^+^ at pH 7), [28] provided positive charges that partially neutralized the Si-O^−^ ionized silanols (pKa ~ 2). In any case, the overall negative charge of the MSN-NH_2_ particles themselves, combined with the restrictions on the diffusion of large biomolecules within the mesoporous structure, are favorable features that make this material less reactive against large and negatively charged DNAs. This may ultimately be helpful in the selective extraction of small miRNA molecules from biological samples. However, it seems apparent that PEG molecules may not be optimum for miRNA adsorption, since they promote an almost neutral potential and reduce internal diffusion of water.

**Figure 2:**
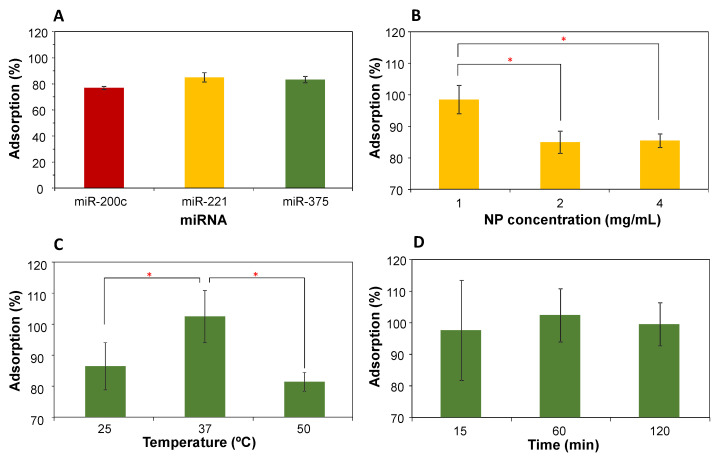
Influence of process parameters on miRNA adsorption on MSN-NH_2_. (**A**) miRNA type. (**B**) Nanoparticle concentration (miR-221). (**C**) Incubation temperature (miR-375). (**D**) Incubation time (miR-375). All experiments were done in triplicate. Data are expressed as the mean ± SD. Statistics: * *p* < 0.05.

The influence of experimental parameters in miRNA corona formation (e.g., miRNA type, particle concentration, temperature and incubation time) was investigated by the adsorption of a single miRNA molecule on MSN-NH_2_. The specific process conditions and results are shown in Figure 2.

With the goal of eventually developing a diagnostic test based on biomolecules circulating in the blood and accessible for simple analysis, the miRNAs selected have been shown to be either up- or downregulated in the serum of prostate cancer patients. The adsorption yield of every miRNA molecule was about 80% (Figure 2A). At this point, because the adsorption behavior of the three miRNA molecules was so similar, we used a single miRNA for each of the remaining experiments. Upon increasing the nanoparticle concentration from 1 mg/mL to 2–4 mg/mL, a significant reduction of miRNA adsorption (miR-221) was observed (*p* < 0.05, Figure 2B), probably due to diffusion limitations in our experimental conditions. Moreover, the incubation temperature was also crucial to optimize miRNA adsorption (miR-375), and the best results were obtained at 37 °C, whereas a significant decrease was reported at lower (25 °C) or higher (50 °C) temperatures (*p* < 0.05, Figure 2C). At lower temperatures the adsorption is thermodynamically unfavorable, while higher temperatures may lead to a degradation of miRNA. In addition, incubation time did not show any influence on the miRNA adsorption (miR-375), at least in the period studied (15–120 min, Figure 2D). This shows that the adsorption process is very fast, consistent with the results from the adsorption of other molecules such as proteins [6].

**Figure 3:**
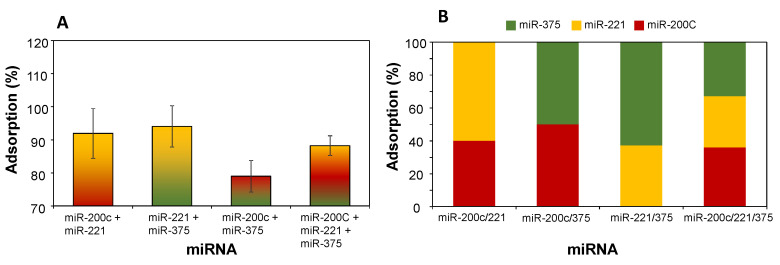
Effect of combined adsorption of various two- and three-components miRNA mixtures on MSN-NH_2_. (**A**) Total miRNA adsorption. (**B**) Distribution of miRNA molecules in the adsorbed mixtures (as determined by PCR analysis). Data are expressed as the mean ± SD.

In this sense, to investigate the potential competition between different miRNA molecules for adsorption into the pores of MSN-NH_2_, we studied the miRNA adsorption process using either two or three miRNAs (Figure 3). The miRNAs were the same as those used above. Three different two-component combinations of miRNAs were analyzed as well as the three-component combination. The total miRNA adsorption showed differences in the range 79–94% (Figure 3A), similar to the adsorption results for single miRNAs. In addition, we used PCR to investigate the relative amounts of each type of miRNA adsorbed onto the MSN-NH_2_. Two different sets of data were processed and plotted (Figure S4 in the Supplementary Information), and the compiled results are presented in Figure 3B. Although there were some small differences in the type of miRNAs adsorbed, none of them were significant. This is consistent with the similar Mw, specific charges, and molecular dimensions of the three miRNAs tested. This indicates that it should be possible to accurately determine the amount of each miRNA from complex solutions when designing a diagnostic test.

**Figure 4:**
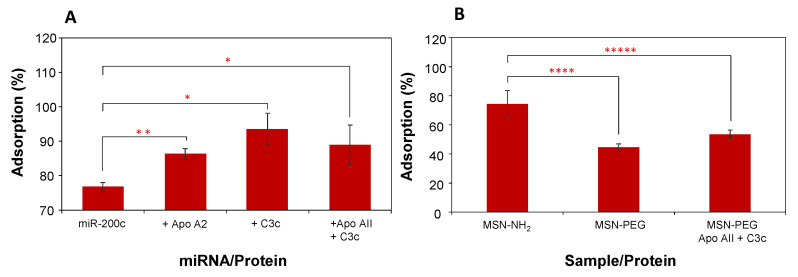
Influence of medium proteins in miRNA adsorption on MSN-NH_2_. (**A**) miR-200c adsorption in the presence of a small protein (Apo AII), a large protein (C3c), or a small and a large protein (Apo AII + C3c). (**B**) Effect of PEGylation of nanoparticle surface on the adsorption of miR-200c in the absence and presence of a small and a large protein (Apo AII + C3c). Data are expressed as the mean ± SD. Statistics: * *p* < 0.05; ** *p* < 0.01; **** *p* < 0.0001; ***** *p* < 0.00001.

In real biological samples, the adsorption of miRNA is part of the overall formation of the biomolecular corona. In this environment, the role of proteins is predominant, as they can promote or hinder the incorporation of miRNA molecules through their interactions. We examined the influence of proteins on miRNA corona formation. It was evaluated in a limited manner by the simultaneous adsorption of miR-200c with either Apolipoprotein AII or Complement 3c. We also performed an additional experiment by adsorbing miR-200c in the presence of both proteins. We chose these two proteins because we have used them in previous studies as representatives of small (Apo-AII) or large (C3C) proteins, and we have shown that protein size is a major determining factor in protein adsorption onto mesoporous silica nanoparticles [12,29]. The results (Figure 4A) indicate that in all cases, the presence of proteins promotes a significant increase of miRNA adsorption on MSN-NH_2_ (AII, *p* < 0.01; C3c, *p* < 0.05; AII + C3c, *p* < 0.05), independently of the protein size. This was formerly pointed out [30], and it is in agreement with the results of Xu et al. [25] who analyzed miRNA from the corona of magnetic nanoparticles after interaction with urine samples. These authors reported that miRNAs can associate with specific proteins of the corona, which promotes their clearance from the liquid medium. In those studies, it was shown that small RNA molecules associate with Argonaute 2-containing protein complexes, avoiding digestion by RNAses in physiological fluids. It seems likely that this interaction is not specific to this combination of miRNA and proteins, but is a general observation of the interactions between proteins and miRNA as the biomolecular corona is formed.

We also investigated the effect of PEGylation of the nanoparticles on the adsorption of miR-200c, both in the absence and presence of AII + C3c proteins. PEGylation of nanoparticles and other surfaces is well known to reduce or prevent protein adsorption. The results in Table 1 and Figure 4B indicate that PEGylation leads to a significant reduction in the porosity of the MSN-NH_2_, in turn reducing the amount of the total surface area available for miRNA adsorption and reducing the amount of adsorbed miRNA (*p* < 0.0001). In addition, PEGylation should reduce miRNA adsorption because of the association of proteins and miRNA. Although adding proteins to the miRNA solution improves miRNA adsorption slightly, the adsorption is still clearly lower than for non-PEGylated nanoparticles (*p* < 0.00001).

**Figure 5:**
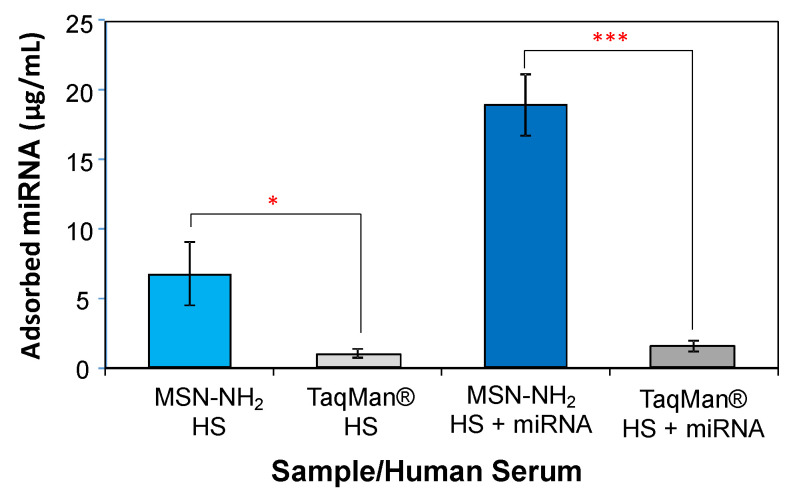
Quantitative comparison of the miRNA isolation performance from human serum samples between the MSN-NH_2_ system and the TaqMan^®^ miRNA ABC purification kit. Standard human serum was used as received (HS) or spiked with the three miRNA PCa biomarkers used in this work (HS + miRNA). Data are expressed as the mean ± SD. Statistics: * *p* < 0.05; *** *p* < 0.001.

Ultimately, any diagnostic test will take place from human serum. To study our ability to detect the upregulation of miRNAs relevant for prostate cancer in human serum, MSN-NH_2_ were incubated with both standard human serum samples and with human serum spiked with the three miRNA PCa biomarkers, and the adsorption of miRNAs from the two experiments were compared. Further, to compare the ability of our system to detect miRNA differences to a common commercial test, the same samples were also processed with the TaqMan^®^ miRNA ABC purification kit. The quantitative results are presented in Figure 5.

It is evident that our system is significantly more efficient for harvesting miRNA moieties from real samples than the commercial kit, which uses iron oxide nanoparticles. Indeed, MSN-NH_2_ were able to collect more than six times as much miRNA from standard human serum as TaqMan^®^ (*p* < 0.05). In addition, the amino functionalized mesoporous silica nanoparticles were able to isolate about 70% of the miRNA spiked in the serum compared to the un-spiked serum, whereas the TaqMan^®^ kit barely showed a difference in the amount of collected nucleic acid (*p* < 0.001). One reason for this difference is likely the additional surface area of the MSNs, and although the surface functionalization of the iron oxide nanoparticles is unknown. Another likely reason is the density of the amino groups on the surface of the MSNs (1.4 mmol/g), which substantially improves the ability of this material to remove miRNA from biological samples.

## 4. Conclusions

In these experiments, our goal was to continue to develop an understanding of the factors that control the formation of the biomolecular corona on mesoporous silica nanoparticles (MSNs) by examining the adsorption of miRNA. We explored the parameters affecting the adsorption process using three miRNAs that are relevant for the future development of a diagnostic test for prostate cancer. Adsorption was optimum with low concentrations of MSN-NH_2_ and at physiological temperature, with 80% of the initial amount of miRNA being adsorbed. The adsorption process was fast, reaching completion in less than 15 min, which is consistent with the results from protein adsorption. While the adsorption of one miRNA did not appear to be affected by another, miRNA adsorption was strongly related to the presence of proteins. Significantly more miRNA was adsorbed when proteins were present than when they were absent, and in addition, preventing protein adsorption by PEGylating the MSN-NH_2_ reduced the amount of adsorbed miRNA. This has important implications in the development of a diagnostic test, where proteins will be a component of the biomolecular corona and will influence the amount and type of adsorbed miRNA. Indeed, we compared the amount of miRNA adsorbed by MSN-NH_2_ to that adsorbed on iron oxide nanoparticles used in the TaqMan^®^ commercial kit, and determined that MSNs MSN-NH_2_ adsorbed six times as much miRNA as the commercial kit. This implies that assays using MSN-NH_2_ may be more sensitive to subtle up- and downregulation of circulating miRNA in the blood of patients than TaqMan^®^, making it more useful in diagnosing the disease state of an individual. Ultimately, this work, combined with proteomic studies, lays the groundwork for the development of MSN-based diagnostic tests.

## Figures and Tables

**Table 1:** Characterization of as-prepared MSN-OH, MSN-NH_2_ and MSN-PEG samples.

Sample	N_2_ Physisorption			Coverage NH_2_		Coverage PEG_4_		Diameter (nm) ^b^	ζ-Potential (mV)
	S_BET_ (m^2^/g)	V_pore_ (cm^3^/g)	d_pore_ (nm)	(mmol/g)	(µmol/m^2^)	(mmol/g)	(µmol/m^2^)		
MSN-OH	1106	1.25	3.9	0	0	0	0	45.0 ± 25.8	−17.5 ± 1.5
MSN-NH_2_	471	0.44	3.1	2.11	4.48 ^a^	0	0	45.4 ± 22.3	−8.9 ± 3.4
MSN-PEG	80	0.34	2.4	1.42	17.75 ^a^	0.69	8.63 ^a^	50.9 ± 29.3	−0.3 ± 0.3

^a^ Surface organic group coverage: µmol/g (from carbon elemental analysis) divided by m^2^/g (from N_2_ physisorption). ^b^ Hydrodynamic diameter determined by DLS.

## Supplementary Materials

The following are available online at https://www.mdpi.com/article/10.3390/nano11051196/s1: Synthesis and characterization of amino functionalized mesoporous silica nanoparticles. TaqMan^®^ miRNA ABC purification kit protocols. Figure S1: DLS results and TEM images. Figure S2: Powder XRD patterns. Figure S3: Nitrogen adsorption isotherms and pore size distribution. Figure S4: qPCR results over adsorption of miRNA mixtures.
