# Supplementary material for: Isolation and Quantification of miRNA from the Biomolecular Corona on Mesoporous Silica Nanoparticles
Source: Nanomaterials (Basel). 2021 May 1;11(5):1196. doi: 10.3390/nano11051196 (PMC8147382; doi:10.3390/nano11051196)
Supplement: Supplementary file 1 [file nanomaterials-11-01196-s001.zip › nanomaterials-1165857-supplementary.pdf]

nanomaterials

Supplementary Materials

Isolation and Quantification of miRNA from the Biomolecular Corona on Mesoporous Silica Nanoparticles

Carla Vidaurre-Agut [1,2], Eva María Rivero-Buceta [1], Christopher C. Landry [3,*] and Pablo Botella [1,*]

[1] Instituto de Tecnología Química, Universitat Politècnica de València-Consejo Superior de Investigaciones Científicas, Avenida de los Naranjos s/n, 46022 Valencia, Spain; carviag@itq.upv.es (C.V.A.); evribu@upvnet.upv.es (E.M.R.B.)
[2] Instituto de Instrumentación para Imagen Molecular (I3M), Centro Mixto CSIC-Universitat Politècnica de València, Camino de Vera s/n, 46022 Valencia, Spain
[3] Department of Chemistry, University of Vermont, 82 University Place, Burlington, VT 05405, USA
* Correspondence: christopher.landry@uvm.edu (C.C.L.); pbotella@itq.upv.es (P.B.)

Contents

1. Synthesis of modified mesoporous silica nanoparticles S2
   1.1. Synthesis of mesoporous silica nanoparticles S2
   1.2. Synthesis of 3-aminopropyl-modified mesoporous silica nanoparticles S2
   1.3. Synthesis of PEGylated mesoporous silica nanoparticles S2
2. Characterization of modified mesoporous silica nanoparticles S2
   2.1. Dynamic light scattering (DLS) S3
   2.2. Powder X-Ray diffraction (XRD) S4
   2.3. Nitrogen Adsorption isotherms S5
3. Isolation and of miRNAs from biomolecular corona S5
   3.1. TaqMan® miRNA ABC purification kit S5
   3.2. qPCR results S8

1. Synthesis of Modified Mesoporous Silica Nanoparticles

1.1. Synthesis of Mesoporous Silica Nanoparticles

1.00 g hexadecyltrimethylammonium bromide (CTAB) was dissolved in 500 mL of NaOH 14 mM at 80 °C with strong stirring. Then, 5.0 mL of tetraethyl orthosilicate (TEOS) was dropped slowly. The obtained gel was 1:0.12:0.31:1245 SiO_2/CTAB/NaOH/H_2O. After 2 h the resulting mixture was cooled in ice, filtered off and washed with water and methanol. The solid was freeze-dried at -55 °C for 16 h.

2.1. Synthesis of 3-Aminopropyl-Modified Mesoporous Silica Nanoparticles

Amine derivatized MSN (MSN-NH_2) were prepared by surface functionalization of MSN-OH with 3-aminopropyltriethoxysilane (APTES). First, 500 mg of MSN-OH were dried at 350 °C and vacuum for 3 h. Subsequently, particles were placed under inert atmosphere and 20 mL of anhydrous toluene was added. The mixture was heated to reflux and 975 µL of APTES (4.2 mmol) was added, leaving the mixture stirring overnight. The obtained product was filtered off, washed with toluene and methanol and freeze-dried (-55 °C, 16 h).

2.3. Synthesis of PEGylated Mesoporous Silica Nanoparticles

PEG-derivatized nanoparticles (MSN-PEG) were prepared by surface functionalization of MSN-NH_2. A total of 200 mg of amino NPs were suspended in 20 mL of anhydrous dichloromethane. Then, 250 µL of diisopropyl amine were injected under nitrogen atmosphere. Subsequently, 150 mg of 2,5,8,11-tetraoxatetradecan-14-oic acid succinimidyl ester (Iris Biotech GMBH) was added. The reaction was stirred overnight at room temperature. Afterwards, the solvent was removed under reduced pressure and the nanoparticles were suspended in 100 mL of ethanol by stirring. Later, the suspension was filtrated ethanol (300 mL). Finally, the material was freeze-dried (-55 °C, 16 h).

2. Characterization of Modified Mesoporous Silica Nanoparticles

Powder X-ray diffraction (XRD) patterns were collected on a Philips X'Pert diffractometer equipped with a graphite monochromator, operating at 40 kV and 45 mA using nickel-filtered Cu Kα radiation (λ = 0.1542 nm). Nitrogen gas adsorption isotherms were measured in a Micromeritics Flowsorb apparatus. Surface area and pore size distribution calculations were carried out using the BET-BJH method. Samples for transmission electron microscopy (TEM) were dispersed in a mixture of EtOH : H_2O (2.5 : 1 v/v) and directly transferred to carbon coated copper grids. TEM micrographs were collected using a JEOL JEM 2100F microscope operating at 200 kV. ζ-potential measurements were conducted in a Zetasizer Nano ZS (Malvern Instruments Ltd., Worcestershire, UK). The dried material was re-suspended in deionized water (pH 7) at a concentration of 5 µg/mL and measurements were performed at 25 °C. The mean hydrodynamic diameter was determined by cumulative analysis. Surface coverage of organic groups (R-NH_2 and R-PEG) was calculated from carbon elemental analysis in a FISONS, EA 1108 CHNS-O equipment, whereas the water content was determined from thermogravimetric (TGA) measurements (Mettler-Toledo TGA/SDTA851e).

2.1. Dynamic Light Scattering (DLS)

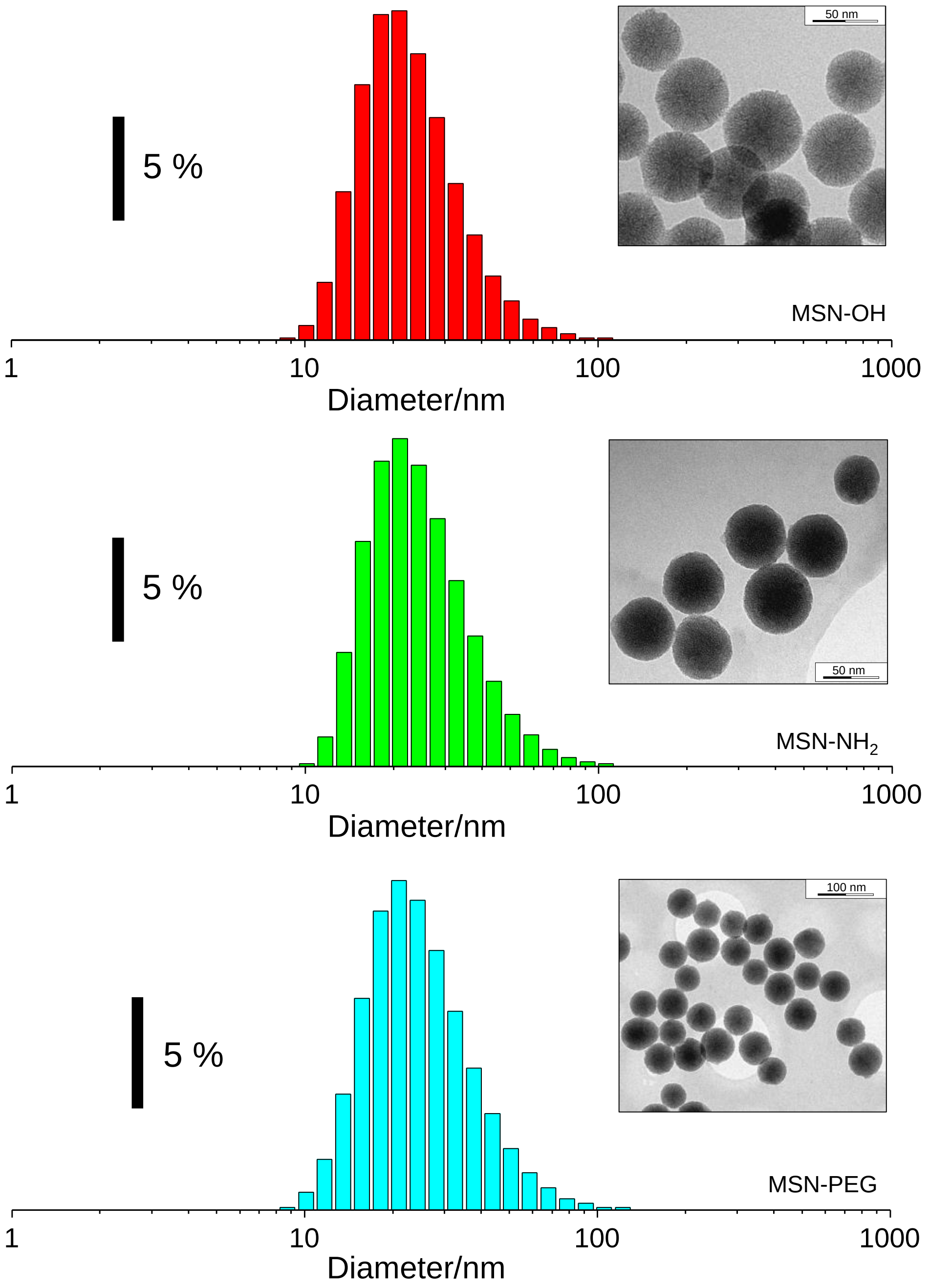

Figure S1. Particle hydrodynamic diameter of as-synthesized materials (log-10 scale) as determined in water by DLS (volume output), and associated representative images (insets). The 5% black bar represents DLS intensity signal percentage.

2.2. Powder X-Ray Diffraction (XRD)

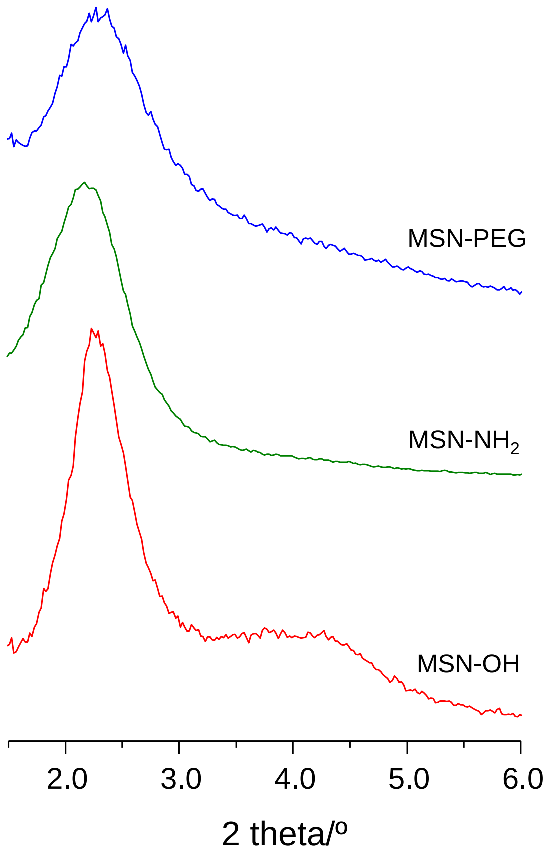

Figure S2. Powder XRD patterns of as-made materials.

2.3. Nitrogen Adsorption Isotherms

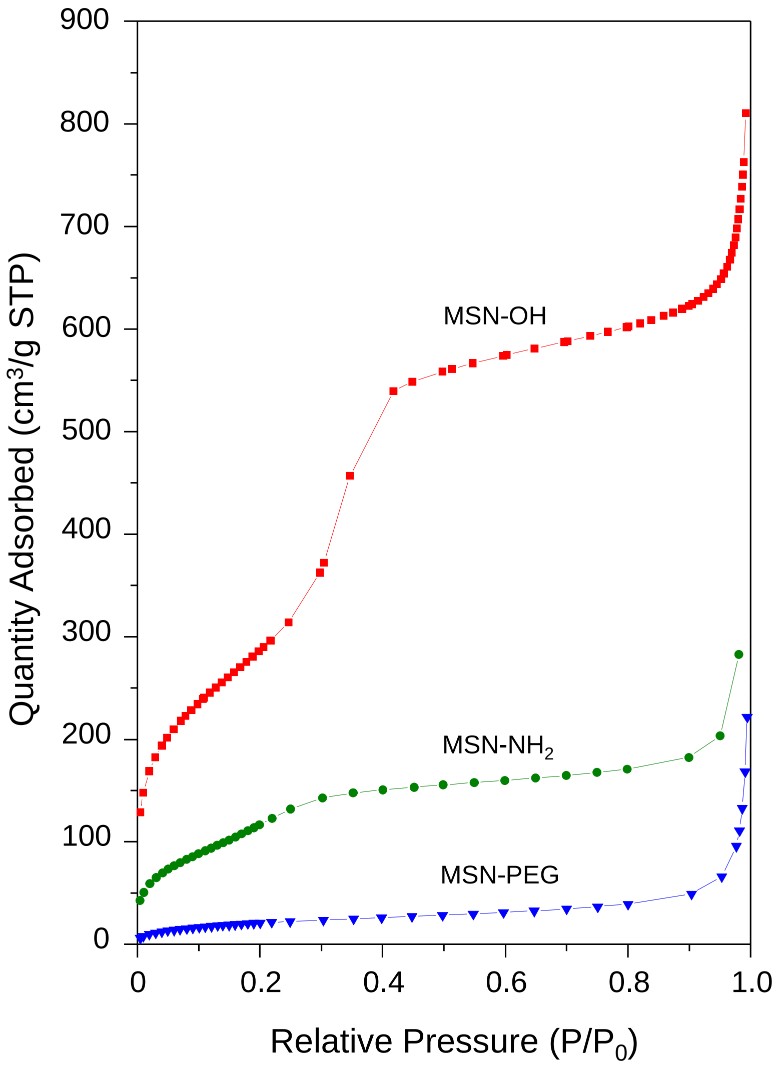

Figure S3. BET nitrogen adsorption isotherms of as-prepared materials.

3. Isolation and of miRNAs From Biomolecular Corona

3.1. TaqMan® miRNA ABC Purification Kit

a) Preparation of the lysates:

1. 250 μL commercial human serum (male AB, Sigma-Aldrich), 250 μL nuclease free water and 5 μL of every miRNA (~0.6 mg/mL solution), were put in a 1.5 mL Eppendorf tube and vortexed for 30 seconds.

2. 50 μL of this soup was added to another 1.5 mL Eppendorf tube and 100 μL ABC buffer was introduced. Then, the mixture was vortexed again for 30 seconds.

b) Prepare the Human Panel Beads:

1. Vortex the beads thoroughly to suspend in solution.

2. Sonicate for 1 minute using an ultrasonic water bath. Mix well by vortexing.

3. Aliquot 80 μL of beads into a 1.5 mL Eppendorf tube for each sample.

4. Place the beads on the magnetic rack for 1 minute and remove the supernatant. Proceed to hybridize the samples.

c) Hybridize the samples:

1. Transfer sample lysates into prepared Human Panel Bead tube(s).

2. Vortex the beads until suspended in solution, then centrifuge briefly (1 min, 14800 rpm).

3. Hybridize for 40 minutes by shaking the tube in a 30 °C Thermomixer® at 1200 rpm.

d) Wash the samples:

1. Place the beads on the magnetic rack for 1 minute and carefully discard the supernatant without disturbing the beads.

2. Add 100 μL wash buffer 1. Vortex briefly, then centrifuge briefly and incubate for 1 minute at room temperature. Place the beads on the magnetic rack for 1 minute and remove the supernatant.

3. Add 100 μL wash buffer 2. Vortex briefly, then centrifuge briefly (1 min, 14800 rpm) and incubate for 1 minute at room temperature. Place the beads on a magnetic rack for 1 minute and remove the supernatant.

4. Repeat step 3 for a third wash.

5. Centrifuge briefly (1 min, 14800 rpm). Place the tube(s) on the magnetic rack for 1 minute. Remove any residual liquid using a fine pipette tip.

e) Elute the samples:

1. Add 100 μL elution buffer and vortex. (1 min, 14800 rpm)

2. Elute for 3 minutes by using a 70 °C Thermomixer® at 1200 rpm.

3. Immediately place the tube(s) on the magnetic rack for 1 minute.

4. Carefully transfer the supernatant containing the miRNA sample(s) into a clean 1.5 mL Eppendorf tube.

5. Centrifuge briefly (1 min, 14800 rpm) and freeze-dry (-55 °C, 16 h).

f) Analyze isolated miRNA.

The miRNA pellet was reconstituted with 10 μL endonuclease free water, and the adsorbed miRNA was determined at 260 nm using a Nanodrop™ ND1000 spectrophotometer. Then, to determine the miRNA distributions in the mixtures, the freeze-dried miRNA suspensions were reconstituted in 100 μL nuclease-free water rotating at 4 °C for 1 h. Concentrations at this stage were determined on a Nanodrop 2000 spectrophotometer (ThermoFisher). Either 150 ng (Group I—same RNA mass) or 2.5 μL (Group II—same RNA volume) RBA was used as input to the miScript RT cDNA synthesis kit (Qiagen) following the manufacturer's recommended protocol. The miSCript SYBR Green PCR kit (Qiagen) was used to interrogate individual miRNA on a ViiA7 (ThermoFisher) following manufacturer's protocol using 0.1 μL cDNA per 10 μL qPCR reaction in a 384-well plate, with triplicate wells for each cDNA/standard. The included Universal Reverse Primer was used with miRNA-specific forward primers:

miR-200c-3p: 5′ – TAATACTGCCGGGTAATGATGGA – 3′

miR-221-3p: 5′ – AGCTACATTGTCTGCTGGGTTTC – 3′

miR-375-3p: 5′ – TTTGTTCGTTCGGCTCGCGTGA – 3′

A 5-point serial dilution (1:10 – 1:10^5) was used to generate a standard curve from a mixture of 2 μL cDNA synthesized (both Groups I and II) from MSN samples incubated with all three miRNAs (six total cDNA samples). Wells with poor amplification and/or melt curves were excluded. Ct values, relative quantity, and standard deviation were calculated in the QuantStudio™ software (ThermoFisher) and plotted in Prism 8 (GraphPad).

Three different two-component miRNA mixtures were analyzed in this manner: miR-200c + miR-221; miR-200c + miR-375 and miR-221 + miR-375. An additional analysis was performed using a three-component miRNA mixture (miR-200c + miR-221 + miR-375). In the three-component adsorption experiment, 1.0 mg MSN-NH_2 was put in a 1.5 mL Eppendorf tube and 5 μL of every miRNA (0.6 mg/mL solution) and 500 μL nuclease free water was added, and the procedure from this point was identical to that for the two-

component mixtures described above. Two different sets of data for every studied mixture were processed and plotted (Figure S1).

3.2. qPCR Results

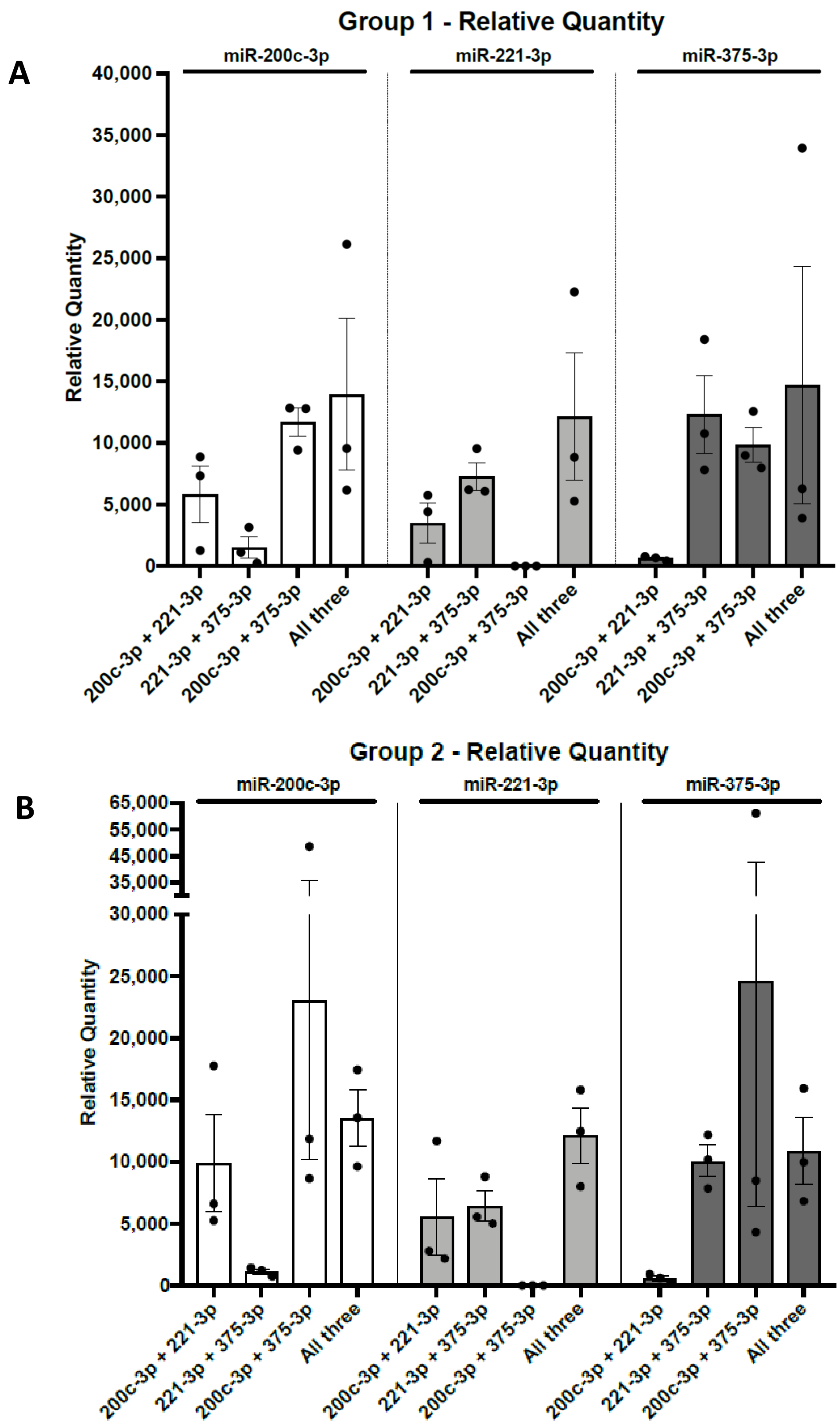

Figure S4. miRNA distribution in the different two- and three-component mixtures adsorbed on MSN-NH_2 (as determined by PCR analysis). Experiments were done in duplicate (Groups 1 and 2). Black circles indicate the different measurements performed on every miRNA mixture.
